# Psychological Stress Exacerbates Inflammation of the Ileum via the Corticotropin-Releasing Hormone-Mast Cell Axis in a Mouse Model of Eosinophilic Enteritis

**DOI:** 10.3390/ijms23158538

**Published:** 2022-08-01

**Authors:** Atsushi Kanamori, Fumio Tanaka, Masaki Ominami, Yuji Nadatani, Shusei Fukunaga, Koji Otani, Shuhei Hosomi, Noriko Kamata, Yasuaki Nagami, Koichi Taira, Yasuhiro Fujiwara

**Affiliations:** 1 Department of Gastroenterology, Graduate School of Medicine, Osaka Metropolitan University, Osaka 545-8585, Japan; atsushi.kanamori@omu.ac.jp (A.K.); ominami@omu.ac.jp (M.O.); dada@omu.ac.jp (Y.N.); shusei@omu.ac.jp (S.F.); kojiotani@omu.ac.jp (K.O.); shuhosomi@omu.ac.jp (S.H.); nkamata@omu.ac.jp (N.K.); yasuaki-75@omu.ac.jp (Y.N.); koichit@omu.ac.jp (K.T.); fujiwara@omu.ac.jp (Y.F.); 2 Department of Gastroenterology, Graduate School of Medicine, Osaka City University, Osaka 545-8585, Japan; 3 Department of Premier Preventive Medicine/MedCity21, Graduate School of Medicine, Osaka Metropolitan University, Osaka 545-8585, Japan

**Keywords:** eosinophilic gastrointestinal disorders, eosinophilic enteritis, eosinophils, mast cells, intestinal permeability, psychological stress, corticotropin-releasing hormone

## Abstract

The effects of psychological stress on eosinophilic gastrointestinal disorders have not been elucidated. This study investigated the effects of psychological stress in a mouse model of eosinophilic enteritis (EoN). BALB/c mice were treated with ovalbumin (OVA) to create an EoN model and subjected to either water avoidance stress (WAS) or sham stress (SS). Microscopic inflammation, eosinophil and mast cell counts, mRNA expression, and protein levels of type 2 helper T cell (Th2) cytokines in the ileum were compared between groups. We evaluated ex vivo intestinal permeability using an Ussing chamber. A corticotropin-releasing hormone type 1 receptor (CRH-R1) antagonist was administered before WAS, and its effects were analyzed. WAS significantly increased diarrhea occurrence and, eosinophil and mast cell counts, and decreased the villus/crypt ratio compared to those in the SS group. The mRNA expression of CRH, interleukin IL-4, IL-5, IL-13, eotaxin-1, and mast cell tryptase β2 significantly increased, and the protein levels of IL-5, IL-13, and OVA-specific immunoglobulin E (IgE) also significantly increased in the WAS group. Moreover, WAS significantly increased the intestinal permeability. The CRH-R1 antagonist significantly inhibited all changes induced by WAS. Psychological stress exacerbated ileal inflammation via the CRH-mast cell axis in an EoN mouse model.

## 1. Introduction

Eosinophilic gastrointestinal disorders (EGIDs) are chronic allergic diseases characterized by eosinophilic inflammation of the gastrointestinal (GI) tract. EGIDs are divided into eosinophilic esophagitis (EoE) and non-esophageal EGIDs, including eosinophilic gastritis (EoG), eosinophilic enteritis (EoN), and eosinophilic colitis (EoC), depending on the involved organs [1]. The most well-studied subgroup is EoE, and the literature on non-esophageal EGIDs is still scarce. The underlying pathophysiology of non-esophageal EGIDs has not been elucidated; however, type 2 helper T cell (Th2)-mediated immune reactions triggered mainly by food antigens play a vital role [2]. Patients with non-esophageal EGIDs have non-specific GI symptoms originating from the GI tract and involving eosinophil infiltration. Treatment of non-esophageal EGIDs includes the systemic administration of glucocorticoids and dietary therapy elimination; however, patients often present a temporal response with later recurrence or treatment resistance [3,4]. The exacerbating factors for non-esophageal EGIDs are still unknown; therefore, detecting these factors is strongly needed.

Similar to EGIDs, other allergic diseases caused by excessive Th2-mediated immune reactions include bronchial asthma, atopic dermatitis, and allergic rhinitis. Previous reports have revealed that psychological stress is a common exacerbating factor for allergic diseases [5,6,7]; however, studies on the association between EGIDs and psychological stress are scarce. Psychological factors such as anxiety and hypersensitivity contribute to esophageal symptoms in patients with EoE [8]. However, there are no previous reports on the effects of psychological stress on non-esophageal EGIDs. Patients with non-esophageal EGIDs sometimes have comorbidities of other allergic diseases, such as atopic dermatitis [9]; hence, we considered that psychological stress might be an exacerbating factor of non-esophageal EGIDs.

Psychological stress increases intestinal permeability in rodents and humans [10], and eosinophils produce the corticotropin-releasing hormone (CRH) in the intestine upon stimulation by psychological stress [11]. CRH activates mast cells and induces intestinal epithelial barrier dysfunction [12]. The increased influx of allergens into the mucosa can result in local immune activation [13]. Patients with EoE present impaired esophageal epithelial barrier function and increased uptake of allergens [14].

Accordingly, we hypothesized that psychological stress exacerbates inflammation via the CRH-mast cell axis in non-esophageal EGIDs. This study aimed to clarify the effects of psychological stress on intestinal permeability and inflammation in a mouse model of oral allergen-induced EoN.

## 2. Results

### 2.1. Establishment of the Oral Allergen-Induced EoN Mouse Model and Effects of Psychological Stress on Diarrhea Occurrence

**Figure 1:**
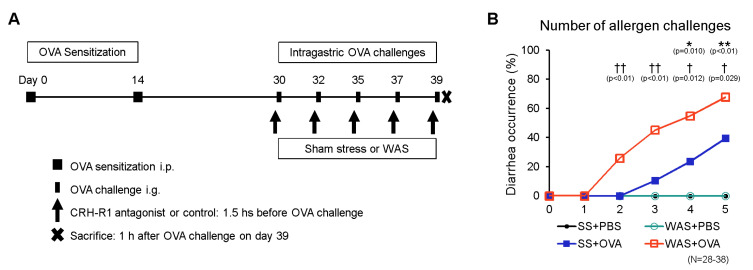
Experimental protocol and diarrhea occurrence. (**A**) The mice were intraperitoneally sensitized on days 0 and 14, and intragastrically challenged using OVA on days 30, 32, 35, 37, and 39. From day 32 to day 39, mice were subjected to WAS or SS for 1 h before OVA or PBS challenge for eight consecutive days. In OVA+WAS-treated mice, CRH-R1 antagonist or vehicle were intraperitoneally administered 1.5 h before each OVA challenge. The mice were sacrificed 1 h after the final OVA challenge, and their ileums were analyzed. (**B**) The comparison of diarrhea occurrence among the four groups of mice: SS + PBS, SS + OVA, WAS + PBS, and WAS + OVA. * *p* < 0.05 and ** *p* < 0.01 between SS + PBS and SS + OVA, † *p* < 0.05 and †† *p* < 0.01 between SS + OVA and WAS + OVA. CRH-R1, corticotropin-releasing hormone type 1 receptor; OVA, ovalbumin; PBS, phosphate buffered saline; SS, sham stress; WAS, water avoidance stress.

We induced EoN in female BALB/c mice with ovalbumin (OVA; grade V; Sigma-Aldrich, St. Louis, MO, USA), and the control mice were challenged with phosphate-buffered saline (PBS), as described in the Materials and Methods section (Figure 1A). The mice were subjected to water avoidance stress (WAS) or sham stress (SS) before the OVA or PBS challenge. The occurrence of diarrhea is presented in Figure 1B. Intragastric OVA challenge in OVA-sensitized BALB/c mice induces antigen-specific diarrhea via a genetically regulated mechanism [15]. SS + OVA-treated mice started developing diarrhea after the third intragastric challenge, and diarrhea occurrence increased with repeated OVA challenges, demonstrating that the allergic mouse model was successfully established. The WAS + OVA-treated mice developed diarrhea after the second intragastric challenge. No diarrhea occurred in the SS group. In contrast, OVA significantly increased the occurrence of diarrhea in the SS group compared to that in the PBS group after the fourth and fifth intragastric challenges (0 vs. 24%, *p* < 0.05; 0 vs. 39%, *p* < 0.01, respectively). In the OVA group, WAS significantly increased the occurrence of diarrhea compared to the SS group after the second, third, fourth, and fifth intragastric challenges (0 vs. 26%, *p* < 0.01; 11 vs. 45%, *p* < 0.01; 24 vs. 55%, *p* < 0.05; 39 vs. 68%, *p* < 0.05, respectively). 

### 2.2. Effects of Psychological Stress on Villous Atrophy and Crypt Hyperplasia

**Figure 2:**
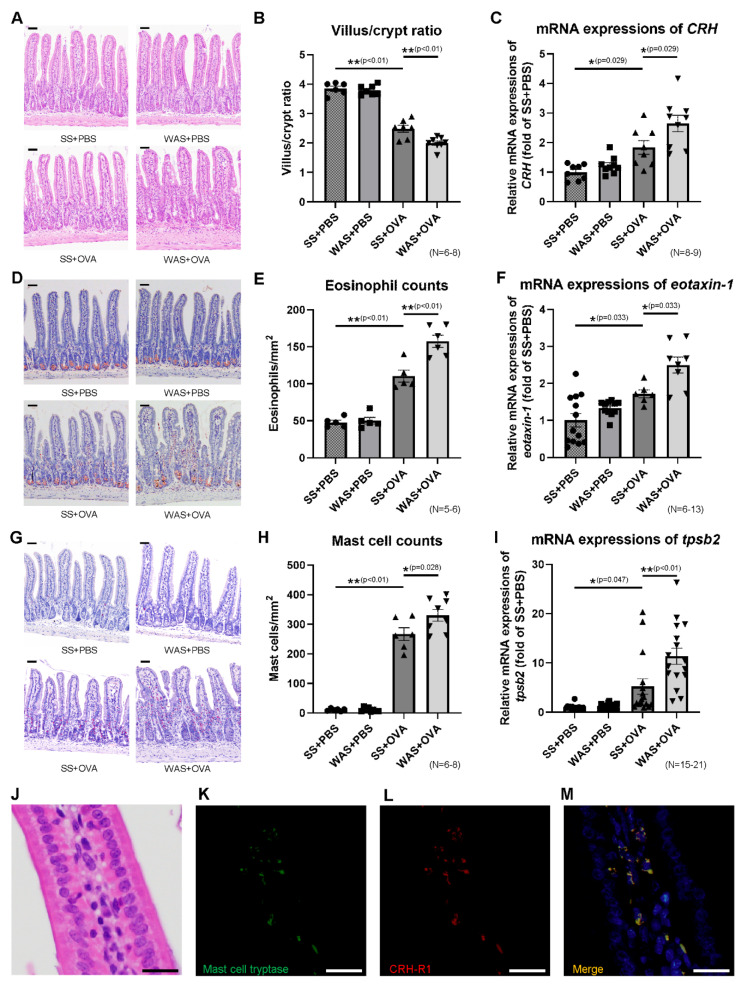
Microscopic findings and mRNA expressions in the ileum. (**A**) Histological images of HE staining. Scale bar = 50 μm. (**B**) The comparison of villus/crypt ratio representing microscopic damage. (**C**) The mRNA expression of CRH. (**D**) DFS staining for eosinophils. Scale bar = 50 μm. (**E**) Eosinophil counts. (**F**) The mRNA expression of eotaxin-1. (**G**) Chloroacetate esterase staining for mast cells. Scale bar = 50 μm. (**H**) Mast cell counts. (**I**) The mRNA expression of tpsb2. (**J**) Histological image of HE staining. (**K**) Mast cell tryptase staining (green). (**L**) CRH-R1 staining (red). (**M**) The colocalization of mast cell tryptase and CRH-R1 is represented in yellow (merge) with blue nuclear counterstain. Each scale bar in Figure 2J–M is 20 μm. * *p* < 0.05, ** *p* < 0.01. CRH, corticotropin-releasing hormone; CRH-R1, corticotropin-releasing hormone type 1 receptor; DFS, direct fast scarlet 4BS; HE, hematoxylin–eosin; tpsb2, tryptase beta 2.

Mice were sacrificed 1 h after the final OVA challenge, as described in the Materials and Methods (Figure 1A). The ileal tissues were isolated to evaluate OVA-induced immune reactions due to the preferential absorption of OVA from the ileum [16]. The villus/crypt ratio is presented in Figure 2A,B. Intestinal eosinophilic inflammation is associated with a reduced villus/crypt ratio [17]. In the SS group, OVA significantly decreased the villus/crypt ratio of the intestinal mucosa by 35% compared with that in the PBS group. In the OVA group, WAS significantly decreased the villus/crypt ratio of the intestinal mucosa by 20% compared with that in the SS group, indicating that WAS exacerbated microscopic inflammation.

### 2.3. Effects of Psychological Stress on the Ileal mRNA Expression of CRH

The mRNA expression of CRH is presented in Figure 2C. In the SS group, OVA significantly increased the intestinal mRNA expression of CRH by 1.6-fold compared to that in the PBS group. In the OVA group, WAS significantly increased the intestinal mRNA expression of CRH by 1.7-fold compared with that in the SS group.

### 2.4. Effects of Psychological Stress on the Numbers of Eosinophils and Mast Cells in Ileal Mucosa

The number of eosinophils in the ileal mucosa of the four groups are presented in Figure 2D,E. In the SS group, OVA significantly increased the number of eosinophils compared to that in the PBS group (47.8 ± 2.9 vs. 110.4 ± 8.1 eosinophils/mm^2^, *p* < 0.01). In the OVA group, WAS significantly increased the number of eosinophils compared to those in the SS group (110.4 ± 8.1 vs. 157.5 ± 8.2/mm^2^, *p* < 0.01). The mRNA expression of eotaxin-1, which plays an important role in eosinophil trafficking, is presented in Figure 2F. In the SS group, OVA significantly increased the ileal mRNA expression of eotaxin-1 by 1.7-fold compared to that in the PBS group. In the OVA group, WAS significantly increased the mRNA expression of eotaxin-1 by 1.5-fold compared to that in the SS group. The numbers of mast cells in the ileal mucosa of the four groups are presented in Figure 2G,H. In the SS group, OVA significantly increased the number of mast cells compared to those in the PBS group (10.9 ± 1.7 vs. 266.9 ± 21.7/mm^2^, *p* < 0.01). In the OVA group, WAS significantly increased the number of mast cells compared to those in the SS group (266.9 ± 21.7 vs. 349.1 ± 20.0/mm^2^, *p* < 0.05). The mRNA expression of tryptase beta 2 (tpsb2), a marker of mast cell activity, is presented in Figure 2I. In the SS group, OVA significantly increased the ileal mRNA expression of tpsb2 by 5.2-fold compared to that in the PBS group. In the OVA group, WAS significantly increased the mRNA expression of tpsb2 by 2.2-fold compared to the SS group.

### 2.5. Mast Cell Tryptase and Corticotropin-Releasing Hormone Type 1 Receptor (CRH-R1) Staining

A histological image of hematoxylin–eosin (HE) staining is presented in Figure 2J, and immunofluorescence staining of mast cell tryptase and corticotropin-releasing hormone type 1 receptor (CRH-R1) is presented in Figure 2K–M. Mast cell tryptase and CRH-R1 were strongly stained in the cytoplasm of mast cells in ileal mucosa. Double immunofluorescence analysis revealed that mast cell tryptase was largely colocalized with CRH-R1 in the ileal mucosa, indicating that mast cells expressed CRH-R1.

### 2.6. Effects of Psychological Stress on the Ileal mRNA Expressions of Th2 Cytokines

**Figure 3:**
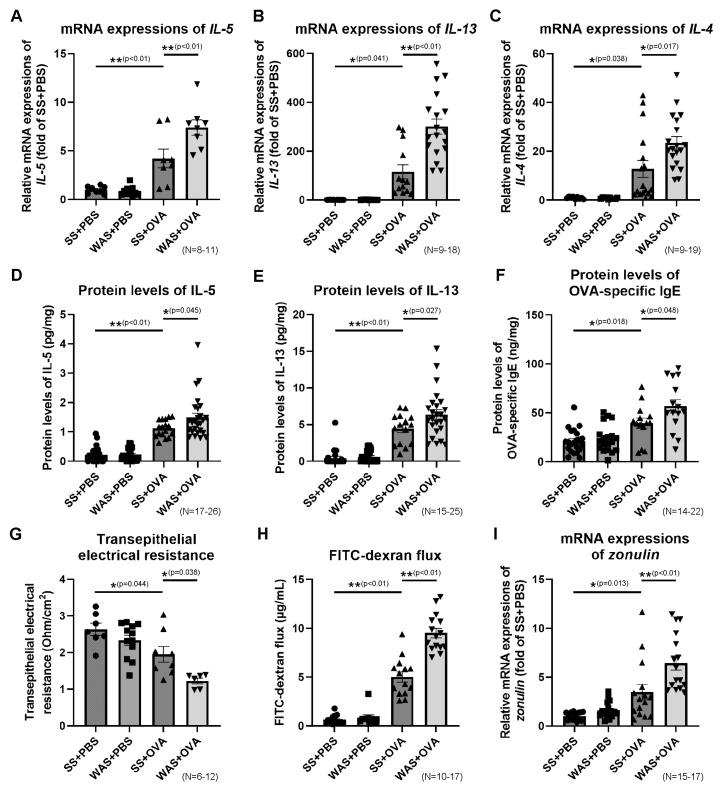
The expressions of Th2 cytokines and OVA-specific IgE in the ileum, and ileal permeability. The mRNA expressions of IL-5 (**A**), IL-13 (**B**), and IL-4 (**C**). Protein levels of IL-5 (**D**), IL-13 (**E**), and OVA-specific IgE (**F**). (**G**) Ex vivo ileal permeability evaluated using TEER. (**H**) Ex vivo ileal permeability evaluated using the FITC-dextran flux. (**I**) mRNA expression of zonulin. * *p* < 0.05, ** *p* < 0.01. FITC, fluorescein isothiocyanate; IgE, immunoglobulin E; IL, interleukin; OVA, ovalbumin; TEER, transepithelial electrical resistance; Th2, type 2 helper T cell.

The mRNA expressions of Th2 cytokines, such as interleukin (IL)-5, IL-13, and IL-4 in the ileum, are presented in Figure 3A–C. In the SS group, OVA significantly increased the mRNA expression of IL-5 by 4.2-fold, IL-13 by 115.7-fold, and IL-4 by 12.8-fold compared to those in the PBS group. In the OVA group, WAS significantly increased the mRNA expression of IL-5 by 1.8-fold, IL-13 by 2.6-fold, and IL-4 by 1.8-fold compared to those in the SS group.

### 2.7. Effects of Psychological Stress on the Protein Levels of Ileal Th2 Cytokines and OVA-Specific Immunoglobulin E (IgE)

The levels of IL-5, IL-13, and OVA-specific immunoglobulin E (IgE) in the ileum are presented in Figure 3D–F. In the SS group, OVA significantly increased the IL-5, IL-13, and OVA-specific IgE levels compared to those in the PBS group (0.2 ± 0.1 vs. 1.1 ± 0.1 pg/mg protein, *p* < 0.01; 0.4 ± 0.2 vs. 4.4 ± 0.5 pg/mg protein, *p* < 0.01; 21.0 ± 2.6 vs. 39.3 ± 5.2 ng/mg protein, *p* < 0.01, respectively). In the OVA group, WAS significantly increased the IL-5, IL-13, and OVA-specific IgE levels compared to those in the SS group (1.1 ± 0.1 vs. 1.5 ± 0.1 pg/mg protein, *p* < 0.05; 4.4 ± 0.5 vs. 6.4 ± 0.6 pg/mg protein, *p* < 0.05; 39.3 ± 5.2 vs. 56.8 ± 6.7 ng/mg protein, *p* < 0.05, respectively). These results indicate that WAS exacerbated Th2 immune reactions at both the mRNA and protein levels.

### 2.8. Effects of Psychological Stress on Intestinal Permeability

Ex vivo intestinal permeability was investigated by measuring the transepithelial electrical resistance (TEER) and fluorescein isothiocyanate (FITC)-dextran (dx) flux. The Ussing chamber experiments revealed a stable transepithelial potential difference after equilibration in the ileal tissues. In the SS group, OVA significantly decreased the TEER by 26% (Figure 3G) and increased FITC-dx flux by 8.0-fold (Figure 3H) in the ileum compared to those in the PBS group. In the OVA group, WAS significantly decreased the TEER by 37% and increased FITC-dx flux by 1.9-fold compared to those in the SS group. The mRNA expression of zonulin, a marker of intestinal permeability, is presented in Figure 3I. In the SS group, OVA significantly increased the mRNA expression of zonulin by 3.5-fold compared to that in the PBS group. In the OVA group, WAS significantly increased the mRNA expression of zonulin by 1.8-fold compared to that in the SS group.

### 2.9. Effects of CRH-R1 Antagonist on Diarrhea Occurrence, Villus/Crypt Ratio, Eosinophils, and Mast Cells in Ileal Mucosa

**Figure 4:**
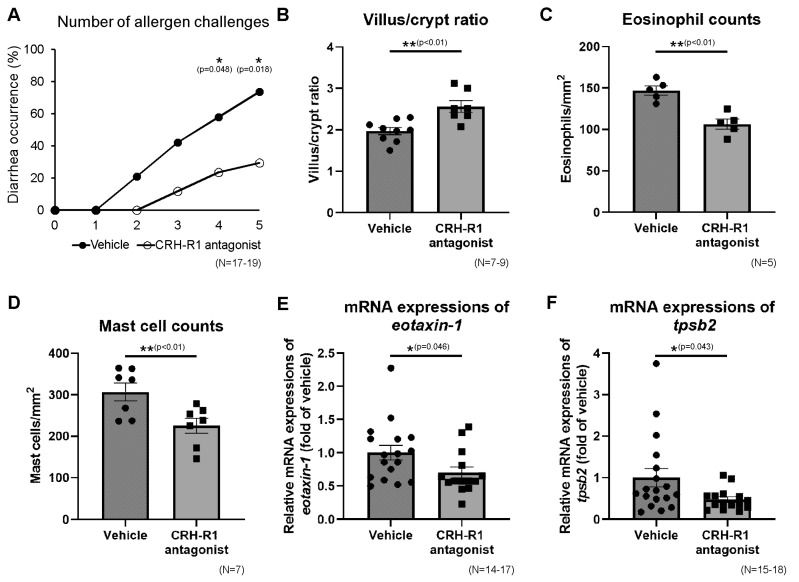
Effects of CRH-R1 antagonist on diarrhea occurrence, microscopic findings, and mRNA expressions in the ileum. Effects of the CRH-R1 antagonist on diarrhea occurrence (**A**), villus/crypt ratio (**B**), number of eosinophils (**C**), and mast cells (**D**) in the ileal mucosa of WAS + OVA-treated mice. Effects of the CRH-R1 antagonist on ileal mRNA expressions of eotaxin-1 (**E**) and tpsb2 (**F**) in WAS + OVA-treated mice. * *p* < 0.05, ** *p* < 0.01. CRH-R1, corticotropin-releasing hormone type 1 receptor; OVA, ovalbumin; tpsb2, tryptase beta 2; WAS, water avoidance stress.

Subsequently, in OVA + WAS-treated mice, Antalarmin (Sigma-Aldrich), CRH-R1 antagonist, or vehicle was administered before WAS, as described in the Materials and Methods section (Figure 1A). Antalarmin significantly decreased the occurrence of diarrhea after the fourth and fifth intragastric challenges compared to the vehicle group (Figure 4A; 24 vs. 59%, *p* < 0.05; 29 vs. 74%, *p* < 0.05, respectively), indicating that the administration of Antalarmin inhibited the effects of WAS. The villus/crypt ratios in the CRH-R1 (−) and CRH-R1 (+) groups under OVA + WAS-treated treatment are presented in Figure 4B. Antalarmin significantly increased the villus/crypt ratio of the intestinal mucosa by 1.3-fold.The number of eosinophils and mast cells in the ileal mucosa of the CRH-R1 (−) and CRH-R1 (+) groups under OVA + WAS-treated conditions are presented in Figure 4C,D. Antalarmin significantly decreased the number of eosinophils and mast cells by 28% and 27%, respectively. Additionally, Antalarmin significantly decreased the ileal mRNA expression of eotaxin-1 and tpsb2 by 30% and 53%, respectively (Figure 4E,F).

### 2.10. Effects of CRH-R1 Antagonist on the Ileal mRNA Expressions of Th2 Cytokines, Protein Levels of Th2 Cytokines and OVA-Specific IgE

**Figure 5:**
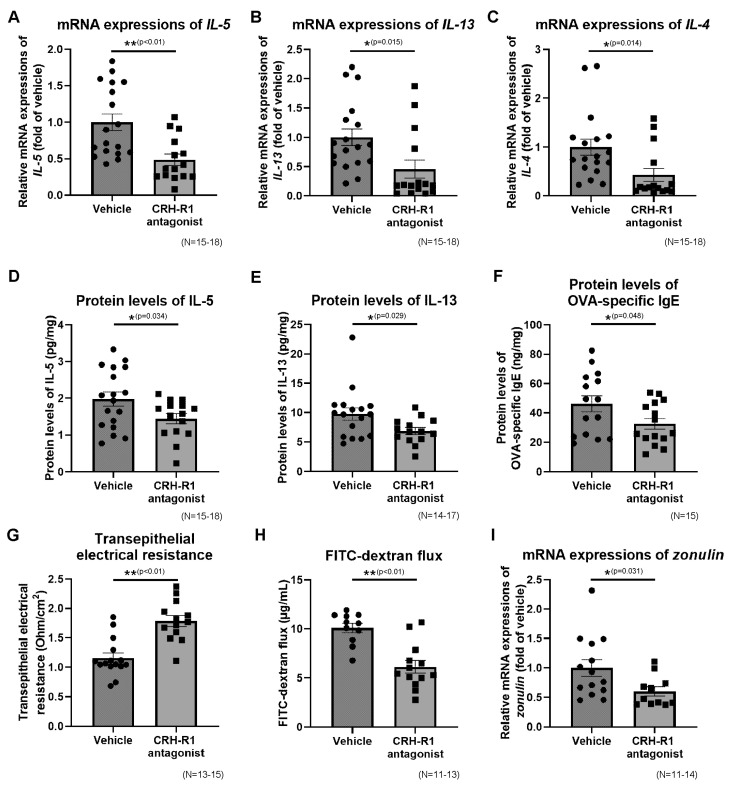
Effects of the CRH-R1 antagonist on Th2 cytokines and OVA-specific IgE in the ileum, and ileal permeability. Effects of the CRH-R1 antagonist on ileal mRNA expressions of IL-5 (**A**), IL-13 (**B**), and IL-4 (**C**) in WAS + OVA-treated mice. Effects of the CRH-R1 antagonist on ileal protein levels of IL-5 (**D**), IL-13 (**E**), and OVA-specific IgE (**F**) in WAS + OVA-treated mice. Effects of CRH-R1 antagonist on TEER (**G**), FITC-dextran flux (**H**), and mRNA expression of zonulin (**I**) in the ileum of WAS + OVA-treated mice. * *p* < 0.05, ** *p* < 0.01. CRH-R1, corticotropin-releasing hormone type 1 receptor; FITC, fluorescein isothiocyanate; IgE, immunoglobulin E; IL, interleukin; OVA, ovalbumin; TEER, transepithelial electrical resistance; Th2, type 2 helper T cell; WAS, water avoidance stress.

The ileal mRNA expressions of IL-5, IL-13, and IL-4 in the CRH-R1 (−) and CRH-R1 (+) groups under OVA + WAS-treated conditions are presented in Figure 5A–C. Antalarmin significantly decreased the mRNA expressions of IL-5, IL-13, and IL-4 by 52, 54, and 56%, respectively. The ileal protein levels of IL-5, IL-13, and OVA-specific IgE in the CRH-R1 (−) and CRH-R1 (+) groups after OVA + WAS-treated conditions are presented in Figure 5D–F. Antalarmin significantly decreased the levels of IL-5, IL-13, and OVA-specific IgE (2.0 ± 0.2 vs. 1.4 ± 0.1 pg/mg protein, *p* < 0.05; 9.8 ± 1.0 vs. 6.9 ± 0.6 pg/mg protein, *p* < 0.05; 46.2 ± 5.5 vs. 32.6 ± 3.7 ng/mg protein, *p* < 0.05, respectively).

### 2.11. Effects of CRH-R1 Antagonist on Intestinal Permeability

Ileal permeability in the CRH-R1 (−) and CRH-R1 (+) group under OVA + WAS-treated conditions are presented in Figure 5G,H. Antalarmin significantly increased the TEER by 1.5-fold and decreased the FITC-dx flux by 39%. Additionally, Antalarmin significantly decreased the ileal mRNA expression of zonulin by 40% (Figure 5I).

## 3. Discussion

As a primary finding of this study, psychological stress exacerbates EoN, including an increased occurrence of diarrhea, microscopic inflammation, eosinophilic and mast cell infiltration, and Th2 cytokines in the ileum, which are associated with increased ileal permeability. We also demonstrated that the administration of a CRH-R1 antagonist significantly inhibited the effects of psychological stress related to ileal inflammation. To our knowledge, this is the first report revealing the involvement of psychological stress via the CRH-mast cell axis regarding the mechanisms of exacerbation of EoN. Psychological stress may be an exacerbating factor in patients with EoN, and CRH may be a novel therapeutic target for treating EoN.

Mast cells are myeloid cells that are widely distributed in the vascular connective tissue. Activation of mast cells by psychological stress may exacerbate diseases derived from the organs in which they are distributed. Psychological stress exacerbates coronary artery disease through the activation of coronary mast cells leading to local inflammation [18]. The degranulation of mast cells induced by CRH under stress conditions leads to disruption of the blood–brain barrier, which plays an important role in neurological diseases, such as Parkinson’s disease and Alzheimer’s disease [19]. In addition, mast cells are particularly abundant at host-environment interfaces, such as the skin and mucosal surfaces of the GI tract. Atopic dermatitis and nasal allergies are exacerbated by psychological stress, and the activation of mast cells by CRH in human skin and nasal mucosa may be a key mechanism [7,20].

CRH is a key physiological mediator in stress response and is a significant regulator of the hypothalamic–pituitary–adrenal axis. In addition to its central effects, CRH in the GI tract has peripheral effects on the immune system under stressful conditions [21,22]. Intestinal immune-related cells, including eosinophils, enterochromaffin cells, macrophages, and T-cells, produce and release CRH upon activation. Among these, most CRH-expressing cells are eosinophils [11]. Stress activates the enteric nervous system, resulting in the release of substance P from nerve endings in the intestine [23], which induces eosinophils to produce CRH. In this study, the intestinal mRNA expression of CRH increased in a mouse model of EoN and with WAS. The intestinal mRNA expression of CRH demonstrated a trend similar to the number of eosinophils, supporting a previous study revealing that intestinal CRH is mainly derived from eosinophils [11].

In this study, double immunofluorescence analysis revealed that mast cell tryptase was largely colocalized with CRH-R1 in the ileal mucosa. This result indicates that CRH receptors are mainly expressed in mast cells [24]. Local CRH activates mast cells via the CRH-R1, which may increase paracellular epithelial permeability [12,25], resulting in an increased influx of protein allergens into the ileal mucosa [24]. OVA, the allergen used in this study, is preferentially absorbed from the ileum via the paracellular and endocytic pathways in animal studies [16]. After OVA sensitization, OVA infiltrates into the mucosa, and crosslinks with allergen-specific IgE antibodies on the surface of mast cells. As a result, mast cells are activated and release various chemical mediators such as tryptase to enhance inflammation. In this study, WAS increased intestinal permeability and OVA-specific IgE levels in the ileum, and a CRH-R1 antagonist inhibited these effects. Psychological stress may increase the uptake of OVA via the CRH-mast cell axis and intestinal permeability. Moreover, increased local OVA-specific IgE levels may indicate increased OVA infiltration into the ileal mucosa.

The mechanism by which OVA uptake triggers EoN is thought to involve Th2-mediated immune reactions, resulting in the secretion of cytokines such as IL-5, IL-4, and IL-13. IL-5, produced by Th2 cells, eosinophils, and mast cells, is a critical growth factor for eosinophils [26]. IL-4 initiates Th2-mediated immune reactions by inducing the conversion of naive T cells to Th2 cells. Additionally, IL-4 promotes the production of IL-13 in mast cells and activates B cell class switching to produce IgE. In this study, WAS increased ileal IL-5, IL-4, and IL-13 levels, and the CRH-R1 antagonist inhibited the effects of WAS on Th2 cytokine production. It has been suggested that psychological stress may increase the uptake of allergens, resulting in enhanced Th2-mediated immune reactions that induce increased diarrhea occurrence, decreased villus/crypt ratio of the intestinal mucosa, and increased eosinophil and mast cell counts. In clinical practice, it is difficult to accurately identify allergens specific to patients with EoN and maintain a thorough elimination dietary therapy. If the uptake of allergens increases by psychological stress in patients with EoN via the same mechanism as in mice, stress reduction and/or CRH-R1 antagonist administration may reduce the frequency of EoN exacerbations. Further studies are needed to clarify the relationship between psychological stress and patients with EoN.

## 4. Materials and Methods

### 4.1. Ethics and Animals

Six-week-old female BALB/c mice, weighing approximately 20 g, were purchased from Japan SLC Inc. (Shizuoka, Japan). All the mice were housed in plastic cages and maintained under a standard environment (12-h light and dark cycle, free feeding). They were randomly assigned to various groups. The experimental protocol was approved by our institution’s Animal Care and Use Committee (Approval no: 20,004).

### 4.2. Mouse Model of Oral Allergen-Induced Intestinal Eosinophilic Inflammation and Psychological Stress

In this study, 7-week-old mice were sensitized and challenged using OVA via the modifying previously reported protocols [15]. Briefly, mice were sensitized by intraperitoneal administration of OVA (50 µg of OVA adsorbed to 1 mg of aluminum hydroxide [Sigma-Aldrich] adjuvant) on days 0 and 14 and intragastrically challenged on days 30, 32, 35, 37, and 39 (10 mg of OVA suspended in 250 µL of PBS) (Figure 1A). Control mice were sensitized using OVA and challenged using phosphate-buffered saline (PBS). From days 32 to 39, the mice were subjected to WAS or SS for 1 h before OVA or PBS challenge, as previously described [27]. During the WAS treatment, each mouse was placed on a circular platform (4 cm in diameter) located at the center of a standard plastic cage (370 × 260 × 330 mm), which was filled with tap water 1 cm below the surface of the platform for 1 h/day and repeated for eight consecutive days. In contrast, each mouse was placed on the same platform in a waterless plastic cage and set free during the SS treatment. The mice were randomly divided into four groups: SS + PBS, WAS + PBS, SS + OVA, and WAS + OVA.

### 4.3. Therapeutic Intervention with the CRH-R1 Antagonist

Antalarmin (Sigma-Aldrich), a CRH receptor type 1 (CRH-R1) antagonist, was diluted with 0.01% Tween 80 as previously described [28]. Subsequently, in OVA + WAS-treated mice, Antalarmin (40 mg/kg) or vehicle was intraperitoneally administered 30 min before WAS on days 30, 32, 35, 37, and 39 (Figure 1A).

### 4.4. Tissue Isolation

The mice were anesthetized using 2% isoflurane and sacrificed 1 h after the final OVA challenge on day 39, followed by isolation of ileal tissues. For ex vivo experiments using the Ussing chamber, the tissues were immediately immersed in ice-cold oxygenated Krebs buffer containing the following (mmol/L):115.0 NaCl, 1.25 CaCl_2_, 1.20 MgCl_2_, 2.0 KH_2_PO_4_, 25.0 NaHCO_3_, and 10.0 C_6_H_12_O_6_ (pH 7.35). Tissue segments were immediately frozen on dry ice and stored at −80 °C for quantitative reverse transcriptase-polymerase chain reaction (qRT-PCR) and protein analysis. For histological evaluation and immunofluorescence staining, the tissues were fixed with 4% paraformaldehyde phosphate buffer solution (Wako Pure Chemical Industries, Ltd., Osaka, Japan.) and embedded in paraffin.

### 4.5. Diarrhea Occurrence

The mice were placed on paper towels in clear plastic cages for 1 h after the PBS/OVA challenge. After 1 h, the wet paper towels were observed for diarrhea, and the occurrence of diarrhea was determined.

### 4.6. Microscopic Inflammation in the Ileum

Tissue segments were stained using HE, and 10 well-oriented crypt-villus units were randomly chosen for villous height and crypt depth measurements at ×10 magnification. Villous height and crypt depth in the ileum were measured using a light microscope (BX53, Olympus, Tokyo, Japan). The results were expressed as the villus/crypt ratio, representing microscopic inflammation.

### 4.7. Ileal Eosinophil and Mast Cell Counts

Tissue segments were stained for eosinophils using a direct fast scarlet 4BS (DFS). They were also stained for mast cells using chloroacetate esterase (Sigma-Aldrich) activity, as previously described [29], and lightly counterstained with hematoxylin. The eosinophils and mast cells were quantified using a light microscope attached to the image analysis system. At least 10 randomly selected areas of the ileal mucosa were counted on each slide at ×20 magnification. Data are expressed as the number of eosinophils or mast cells per mm^2^ of ileal mucosa.

### 4.8. Immunofluorescence for Mast Cell Tryptase and CRH-R1

The slides were deparaffinized, and heat-induced antigen retrieval was performed using Histofine (Nichirei Biosciences Inc., Tokyo, Japan). Slides were washed with Tris-buffered saline containing Tween 20 (3 × 5 min) and incubated with 10% donkey serum for 60 min. Primary antibodies or FITC-conjugated anti-mouse mast cell tryptase antibodies (1:2000, mouse monoclonal, ab2378; Abcam, Cambridge, UK) were applied to the sections and incubated overnight at 4 °C. The primary antibody used was anti-CRHR1 antibody (1:1000, goat polyclonal, ab77686; Abcam, Cambridge, UK). For double immunofluorescence labeling, we used the primary and FITC-conjugated antibodies described above, and the appropriate secondary antibody (donkey anti-goat IgG) labeled with Alexa Fluor 594 (1:1000, ab150136; Abcam). Following incubation with the antibodies, the tissues were washed with Tris-buffered saline containing Tween 20 (3 × 5 min). The slides were coverslipped with Prolong-Diamond Antifade Mountant with DAPI (Thermo Fisher Scientific, Rockford, IL, USA). Fluorescent images were obtained at the Osaka City University Graduate School of Medicine Core Imaging Facility. The tissues were examined at 400× magnification using a fluorescence microscope (BX53, Olympus).

### 4.9. Quantitative Reverse Transcription-PCR Gene Expression in the Ileum

The local mRNA expression in the ileum was measured. Total RNA was isolated from the ileal tissue using the ISOGEN kit (Nippon Gene, Tokyo, Japan) according to the manufacturer’s protocol. Complementary DNA was produced using the high-capacity RNA-to-cDNA Kit (Life Technologies, Carlsbad, CA, USA), according to the manufacturer’s protocol. qRT-PCR analyses were performed using an Applied Biosystems 7500 Fast Real-Time PCR System and Software (Life Technologies). The reaction mixture was prepared according to the manufacturer’s protocol using the TaqMan Fast Universal PCR Master Mix (Life Technologies). The thermal cycling conditions were as follows: 40 cycles at 95 °C for 15 s and 60 °C for 1 min. The expression levels of CRH, eotaxin-1, tpsb2, IL-5, IL-13, IL-4, and zonulin were quantified and standardized using the TaqMan glyceraldehyde-3-phosphate dehydrogenase (GAPDH; Life Technologies) mRNA levels. The expression levels of these mRNAs are indicated as ratios relative to the mean value for the SS + PBS group. Primers and probes for CRH (Mm01293920_s1, Foster City, CA, USA), eotaxin-1 (Mm00441238_m1), tpsb2 (Mm01301240_g1), IL-5 (Mm00439646_m1), IL-13 (Mm00434204_m1), IL-4 (Mm00445259_m1), and zonulin (Mm00516884_m1) were purchased from Applied Biosystems.

### 4.10. Enzyme-Linked Immunosorbent Assay

Intestinal tissue was homogenized in 2.0 mL of TNE buffer, and supernatants were obtained by centrifugation (10,000 rpm for 10 min) and frozen at −80 ℃ in polypropylene tubes until assayed. IL-5 and IL-13 levels in the ileum were measured via an enzyme-linked immunosorbent assay (ELISA) using immunoassay kits (R&D Systems, Minneapolis, MN, USA), according to the manufacturer’s instructions. OVA-specific IgE levels in the intestinal mucosa were measured using ELISA kits (MD Bioproducts, Saint Paul, MN, USA) according to the manufacturer’s instructions. Intestinal tissue protein levels were quantified using the BCA protein assay (Pierce, Rockford, IL, USA).

### 4.11. Measurement of Ex Vivo Intestinal Permeability, Method 1: Transepithelial Electrical Resistance

Ileal tissues were opened along the mesenteric border, and the tissue segments were mounted in Ussing chambers (Warner Instruments, Hamden, CT, USA) with an aperture of 16.8 mm^2^. Subsequently, TEER was measured to evaluate ex vivo intestinal permeability as previously described [27]. The exposed tissues were bathed in identical Krebs buffer formulations and continuously perfused using 95% O_2_ and 5% CO_2_, while the bath temperature was maintained at 37 °C using a heating circulator (AS ONE, Osaka, Japan). Ileal tissues within the Ussing chambers were equilibrated in oxygenated Krebs buffer at 37 °C for 30 min, and the potential difference (PD) and short-circuit current (Isc) across the tissues were monitored. Two voltage sensing and agar-salt bridge electrodes on either side of the tissue monitored the PD across the tissues. In contrast, two Ag-AgCl current-passing electrodes injected current into the system to maintain a PD of zero. Potential differences and Isc were measured using a VCC 600 single-channel voltage-current clamp (Physiologic Instruments, San Diego, CA, USA). Stable baseline values for PD and Isc confirmed the viability of tissue preparations. Stable baseline values for PD and Isc confirmed the viability of tissue preparations. Following equilibration (30 min), the TEER across the tissues was calculated from Ohm’s law, using the PD and Isc values measured after 30 min.

### 4.12. Measurement of Ex Vivo Intestinal Permeability, Method 2: Macromolecular Transport

Macromolecular transport was assessed by measuring the flux of FITC-dx (molecular weight −4000 Da, Sigma-Aldrich) from the mucosal to the serosal sides of the ileal tissues within the Ussing chamber, which represented paracellular permeability, as previously described [27]. A colorimetric endpoint assay was performed to quantify the amount of FITC in each sample. The FITC concentration of each sample was calculated using a standard curve. The FITC concentration of each sample was determined using a standard curve, and FITC-dx flux was expressed as ngFITC/h/mm^2^.

### 4.13. Statistical Analysis

Data were expressed as the mean ± standard error (SE) for continuous variables and frequency for categorical variables. For categorical data, comparisons between two groups were performed using Fisher’s test, and comparisons between four groups were performed using the χ^2^ test. In contrast, continuous data were compared using Student’s *t*-test or one-way ANOVA test followed by Tukey’s post hoc analysis. Figures were generated using GraphPad Prism (GraphPad Software, San Diego, CA, USA, ver. 9.4.0). All statistical analyses were performed using EZR (Saitama Medical Center, Jichi Medical University, Saitama, Japan, version 1.37), a graphical user interface for R (The R Foundation for Statistical Computing, ver. 3.5.2). The statistical significance was set at a *p*-value of 0.05.

## 5. Conclusions

In conclusion, psychological stress exacerbates ileal inflammation via the CRH-mast cell axis in a mouse model of EoN. Increased intestinal permeability and further influx of allergens into the mucosa may also be implicated in the mechanisms underlying this process.

## Data Availability

All data are available on request from the authors.
